# Lymphatic filariasis transmission in Rufiji District, southeastern Tanzania: infection status of the human population and mosquito vectors after twelve rounds of mass drug administration

**DOI:** 10.1186/s13071-018-3156-2

**Published:** 2018-11-13

**Authors:** Clarer Jones, Billy Ngasala, Yahya A. Derua, Donath Tarimo, Lisa Reimer, Moses Bockarie, Mwelecele N. Malecela

**Affiliations:** 1 Department of Parasitology and Medical Entomology, Muhimbili University of Health and Allied Sciences, P. O. Box 65011, Dar es Salaam, Tanzania; 2 Department of Women’s and Children’s Health International Maternal and Child Health (IMCH), Uppsala Universitet, Uppsala, Sweden; 3 National Institute for Medical Research, P. O. Box 9653, Dar es Salaam, Tanzania; 4 Liverpool School of Tropical Medicine, Liverpool, UK; 5 European and Development Countries Clinical Trials Partnership, Cape Town, South Africa; 6 WHO Regional Office for Africa, Brazzaville, Republic of the Congo

**Keywords:** Lymphatic filariasis, Mass drug administration, Microfilaremia, Circulating filarial antigens, Hydrocele, Elephantiasis, *Wuchereria bancrofti*, Tanzania

## Abstract

**Background:**

Control of lymphatic filariasis (LF) in most of the sub-Saharan African countries is based on annual mass drug administration (MDA) using a combination of ivermectin and albendazole. Monitoring the impact of this intervention is crucial for measuring the success of the LF elimination programmes. This study assessed the status of LF infection in Rufiji district, southeastern Tanzania after twelve rounds of MDA.

**Methods:**

Community members aged between 10 and 79 years were examined for *Wuchereria bancrofti* circulating filarial antigens (CFA) using immunochromatographic test cards (ICTs) and antigen-positive individuals were screened for microfilaraemia. All study participants were examined for clinical manifestation of LF and interviewed for drug uptake during MDA rounds. Filarial mosquito vectors were collected indoor and outdoor and examined for infection with *W. bancrofti* using a microscope and quantitative real-time polymerase chain reaction (qPCR) techniques.

**Results:**

Out of 854 participants tested, nine (1.1%) were positive for CFA and one (0.1%) was found to be microfilaraemic. The prevalence of hydrocele and elephantiasis was 4.8% and 2.9%, respectively. Surveyed drug uptake rates were high, with 70.5% of the respondents reporting having swallowed the drugs in the 2014 MDA round (about seven months before this study). Further, 82.7% of the respondents reported having swallowed the drugs at least once since the inception of MDA programme in 2000. Of the 1054 filarial vectors caught indoors and dissected to detect *W. bancrofti* infection none was found to be infected. Moreover, analysis by qPCR of 1092 pools of gravid *Culex quinquefasciatus* collected outdoors resulted in an estimated infection rate of 0.1%. None of the filarial vectors tested with qPCR were found to be infective.

**Conclusion:**

Analysis of indices of LF infection in the human population and filarial mosquito vectors indicated a substantial decline in the prevalence of LF and other transmission indices, suggesting that local transmission was extremely low if occurring at all in the study areas. We, therefore, recommend a formal transmission assessment survey (TAS) to be conducted in the study areas to make an informed decision on whether Rufiji District satisfied WHO criteria for stopping MDA.

## Background

Lymphatic filariasis (LF) is the second most common vector-borne parasitic disease after malaria and is found in over 83 tropical and subtropical countries [1]. It is estimated that one billion people are at risk of infection and 120 million are affected by LF. Of those at risk of infection, 65% reside in the Southeast Asia, 30% in Africa, and the remainder live in other parts of the tropical world [2, 3]. In sub-Saharan Africa, LF is caused by infection with the parasitic nematode *Wuchereria bancrofti* and transmitted by night-biting mosquitoes of the genera *Anopheles* and *Culex* [4]. The most important filarial vectors in sub-Saharan Africa are *Culex quinquefasciatus* (widespread in urban and semi-urban areas) and members of the *Anopheles gambiae* complex and *An. funestus* group found in rural areas [5].

In 1997, the World Health Assembly passed a resolution calling for the elimination of LF as a public health problem globally [6, 7]. As a result, the Global Programme to Eliminate Lymphatic Filariasis (GPELF) was launched in 2000 by the World Health Organization (WHO). The programme had a principle objective of interrupting the transmission of *W. bancrofti* and *Brugia malayi* through the application of annual mass drug administration (MDA) to the entire at-risk population as well as management and prevention of LF-related disabilities [8]. The fundamental assumption behind the MDA approach is that once the community has been treated long enough, levels of microfilariae will remain below that required to sustain transmission [9]. It has been suggested that after four to six consecutive rounds of MDA, microfilariae load in the endemic population is expected to fall below 1%, and this reduction in microfilaraemia will lead to the reduced acquisition of new infections [10].

LF is endemic in many parts of mainland Tanzania where nearly 70% of the population is at risk, and six million people are estimated to have disabilities due to the disease [11]. LF endemicity varies across the country, with high antigenemia levels of 45–60% along the coast to 2–4% in western Tanzania [11]. Tanzania was one of the first countries in sub-Saharan Africa to adopt the WHO recommended strategy of applying annual MDA to eliminate LF, and the Tanzanian National Lymphatic Filariasis Elimination Programme (NLFEP) was launched on Mafia Island in 2000 [12]. Since then, the programme has expanded to cover a total population of 13 million people treated at least once with ivermectin and albendazole, with a target to extend the coverage to the entire estimated at-risk population of around 34 million people [11].

Monitoring of the transmission dynamics during MDA implementation is essential for measuring the progress and defining MDA endpoints. The main measures used to assess the impact of LF MDA have relied upon detection of parasites, parasite antigens, parasite DNA and/or anti-parasite antibodies in humans [13]. However, detecting these indicators of LF infection in humans are not always definitive of local transmission as they may not account for immigration of infected individuals to areas where transmission has been controlled [13]. On the other hand, detection of filarial parasites in mosquito vectors indicates infection uptake from human hosts while the presence of infective third-stage larvae (L3) indicates a possibility of local transmission [14, 15]. Thus, monitoring *W. bancrofti* infection in humans together with vector infectivity provides a more sensitive measure of local transmission. The present study monitored *W. bancrofti* infection status of the human population and mosquito vectors after twelve annual rounds of MDA with a combination of ivermectin (150–200 mg/kg) and albendazole (400 mg) delivered to individuals aged five years and above in the highly endemic area of southeastern Tanzania.

## Methods

### Study area

This study was conducted in Rufiji District (7°57'S, 38°43'E) southeastern Tanzania about 150 km south of Dar es Salaam. Based on 2012 census data, the district had a total population of 217,274 people [16]. The district lies within the floodplain of Rufiji River, *c.*500 m above sea level. The district has two main rain seasons; a long rainy season between February and May and a shorter less intense one from October to December. The annual rainfall ranges between 800–1000 mm. Rufiji District was purposively selected for the study due to its history of high LF prevalence before the start of elimination activities based on ivermectin and albendazole MDA in 2002. Twelve rounds of annual MDA were completed in Rufiji District between 2002 and 2014 (Table 1).

**Table 1:** Reported treatment coverage during ivermectin and albendazole MDA intervention in Rufiji District, southeastern Tanzania

MDA No.	Treatment year	Target population	Treated population (% coverage)
1	2002	138,370	75,135 (54.3)
2	2003	141,628	123,216 (87.0)
3	2004	144,962	136,267 (94.0)
	2005^a^	–	–
4	2006	151,868	85,046 (56.0)
5	2007	155,443	116,583 (75.0)
6	2008	159,103	115,350 (72.5)
7	2009	162,848	130,279 (80.0)
8	2010	166,682	113,677 (68.2)
9	2011	170,606	150,133 (88.0)
10	2012	236,614	146,060 (61.7)
11	2013	243,835	149,163 (61.2)
12	2014	217,274	168,930 (77.7)

^a^MDA was not implemented in 2005

The district has a total of 83 registered villages across 19 administrative wards. Using stratified random sampling, five villages (Bungu, Nyambili, Nyanjati, Mchukwi and Nyamisati) were selected for the study representing three main ecological settings of the district. Nyamisati is located along the coast of the Indian Ocean while both Bungu and Nyanjati are inland villages with high and low population, respectively. Nyambili is a lowland village at the basin of Rufiji River prone to flooding during the rainy season, and Mchukwi is a relatively highland village. Before MDA in 2000, the prevalence of *W. bancrofti* circulating filarial antigens (detected using immunochromatographic test (ICT) cards), microfilaraemia, hydrocele, and elephantiasis were 49, 18, 12 and 4%, respectively (Tanzanian Ministry of Health and Social Welfare (MoHSW) 2000, unpublished). The present study was conducted in April 2015, about seven months after the 12th annual round of MDA based on a combination of ivermectin and albendazole delivered in September 2014.

### Study population and sampling

Study participants were selected from a list of names of heads of households obtained from the respective village executive officers. Forty heads of households were randomly selected from the list, and all members of the household included for the survey. In 2012, the average household size in Rufiji was reported to be five individuals [16], and thus it was estimated to screen 200 individuals from each village. On the survey date, participants were invited to a central place (dispensary or school premises) for blood testing and interviews. Participants who did not turn up for screening in the central place were followed at their homes.

### LF prevalence, burden and MDA compliance

Consented community members aged between 10 and 79 years) in the study villages were examined for *W. bancrofti* CFA using immunochromatographic test (ICT) cards (Binax Now® Filariasis, Inverness Medical Innovations Inc., Massachusetts, USA) following the manufacturer’s instructions. Briefly, 100 μl of finger-prick blood was collected from each participant and applied to the test card. Test results were read after precisely 10 min as positive, negative or undetermined. All CFA positive cases detected during the daytime were further examined for microfilaria (MF) by using counting chamber technique [17]. For MF testing, 100 μl of finger-prick blood was collected with sterile heparinised capillary tubes starting from 22:00 to 00:00 h and transferred to sample tubes with 900 μl of 3% acetic acid. In the laboratory, the samples were transferred into the counting chamber and examined for MF under a compound microscope. Moreover, participants were examined in privacy by the study clinicians for clinical manifestation of LF. Using pre-designed forms, demographic information such as sex, age, marital status, occupation, and duration of residence in Rufiji were recorded for all participants. Moreover, participants were interviewed on whether they had swallowed ivermectin and albendazole distributed in the community in September 2014 and also their participation in drug uptake in any of the previous 12 annual MDA campaigns.

### Vector and transmission surveys

Filarial mosquito vectors were collected from 10 selected houses from each village using Centre for Disease Control (CDC) light traps (John W. Hock Company, Gainesville, USA). To optimize trap yield, households with thatched roof and open eaves were selected from different parts of each of the trapping village in a non-random approach. Sleeping rooms with a bed and a willing occupant was selected for trapping. CDC light trap was placed beside an occupied bed (with occupant protected with a long-lasting insecticide-treated net) in each household for two consecutive nights. Light traps were switched on between 17:00 and 18:00 h and then turned off between 06:00 and 07:00 h the following morning. Mosquitoes collected from each light trap type were transferred separately into labeled paper cups covered with a netting material and offered cotton pads soaked in a 10% glucose solution and transported to the field laboratory. In the laboratory, they were knocked down with chloroform, sorted, and then identified based on morphological characters. Freshly-killed female filarial mosquito vectors were dissected and examined under a microscope for parity and infection with *W. bancrofti* as described previously [18].

CDC gravid traps were also set outdoors (in the same households used for setting a light trap) to collect gravid mosquitoes for two consecutive nights in each house. The traps were set in peri-domestic areas, and the trappings were conducted as described by Irish et al. [19]. In brief, the traps were switched on between 17:00 and 18:00 h and switched off the next morning between 06:00 and 7:00 h. Collected mosquitoes were treated as described for light trap catch except that the filarial vectors from gravid traps were preserved individually in Eppendorf tubes containing silica gel desiccants for later detection of infection using the quantitative real-time polymerase chain reaction (qPCR) technique.

For qPCR processing, gravid *Cx. quinquefasciatus* were dissected to separate head, thorax, and abdomen. Combined thorax and abdomen segments were processed separately from the heads in pools of five mosquitoes. DNA was extracted from pooled mosquito segments by using the Livak method [20]. In brief, mosquitoes were homogenized in Livak buffer (0.5% sodium dodecyl sulphate (SDS), 0.08M sodium chloride (NaCl), 0.16M sucrose, 0.5M ethylenediaminetetraacetic acid (EDTA) and 0.12M Tris-HCl), proteins and debris separated with 8M potassium acetate, and DNA precipitated with ethanol. The resultant DNA was rinsed in 70% cold ethanol, dried and re-suspended in tris-acetate-EDTA (TAE) buffer. An aliquot of extracted DNA from heads was combined with those from the thorax and abdomen segments and screened in the initial qPCR run to detect infection with *W. bancrofti*. After that, the heads of the positive pools were re-tested in the follow-up qPCR screening to establish whether the positive signal from the initial qPCR run was obtained from the head segment.

The extracted DNA was analysed for the presence of *W. bancrofti* DNA by qPCR using the method of Rao et al. [15]. In brief, the long DNA repeat (LDR) of *W. bancrofti* was targeted with specific forward and reverse primers (LDR1 and LDR2) and *Taq*Man probe for amplification and detection. In the reaction mixture, each 10 μl of PCR consisted of 0.5 μM of each of the two primers (LDR1 and LDR2), 12.5 μl of *Taq*Man probe (Applied Biosystems, Foster City, CA, USA), 1:1 SensiMix™ (Bioline, Meridian Bioscience Asia Pte Ltd, Queenstown, Singapore) and 1 μl of DNA extract. Thermal cycling conditions included 95 °C for 10 min followed by 40 cycles of denaturation at 92 °C for 15 s and annealing at 60 °C for 60 s. Each batch of samples was run with positive and negative controls. Thermal cycling and sample analysis were performed with Agilent MX 3005P qPCR systems, with MXPro software (5301 Stevens Creek Blvd, Santa Clara, CA 95051, USA).

### Data analysis

Data were entered in Excel spreadsheets (Microsoft Corp., Redmond, WA, USA) and subsequently analyzed with SPSS version 20.0 (SPSS, Inc., Chicago, IL, USA). Blood test results for CFA and MF, the presence of hydrocele or elephantiasis, demographic characteristics, and MDA compliance were compared by using the Chi-square test and *P*-values ≤ 0.05 were considered statistically significant. The lower and upper limits of the 95% confidence interval for the prevalence of MF and CFA were calculated according to the method described by Newcombe [21]. For pool screening qPCR, the probability that any one mosquito in the pool was infected with *W. bancrofti* was estimated by pool-screen v2.0.2 software providing maximum likelihood estimates for infection rates as previously described [22].

## Results

### LF prevalence and disease burden

A total of 854 individuals above five years-old in five villages of Rufiji District were examined for CFA, MF, hydrocele and elephantiasis. The overall male to female ratio was 1.3, and the mean age of the participants was 32.3 years (range of 10–79 years). Of 854 individuals screened for LF infection, 9 (1.1%) and 1 (0.1%) had CFA and MF, respectively (Table 2). Males were significantly more infected (CFA) than females (*χ*^2^ = 3.921, *df* = 1, *P* = 0.048). Of the 48 individuals found with the chronic clinical manifestation of LF, 23/481 (4.8%) and 25/854 (2.9%) had hydrocele and elephantiasis, respectively (Table 2). None of the individuals with hydrocele or elephantiasis were found to have MF or CFA.

**Table 2:** Prevalence of CFA, microfilariae and clinical manifestation of lymphatic filariasis infection by gender, age and village location in Rufiji District, southeastern Tanzania

Characteristic	No. (%) examined	No. (%) with CFA [95% CI]	No. (%) with MF [95% CI]	No. (%) with hydrocele^a^	No. (%) with elephantiasis
Gender					
Male	481 (56.3)	8 (1.7) [0.8–3.2]	1 (0.2) [0.0–1.2]	23 (4.8)	18 (3.7)
Female	373 (43.7)	1 (0.3) [0.1–1.5]	0 (0.0) [0.0–1.0]	–	7 (1.9)
Total	854	9 (1.1) [0.6–2.0]	1 (0.1) [0.0–0.7]	23 (4.8)	25 (2.9)
Age (years)					
5–14	188 (22.1)	1 (0.5) [0.1–3.0]	0 (0.0) [0.0–2.0]	0 (0.0)	1 (1.0)
15–34	307 (35.9)	4 (1.3) [0.5–3.3]	1 (0.3) [0.1–1.8]	8 (4.7)	13 (7.8)
≥ 35	359 (42.0)	4 (1.1) [0.4–2.8]	0 (0.0) [0.0–1.1]	15 (7.0)	11 (5.2)
Total	854	9 (1.1) [0.6–2.0]	1 (0.1) [0.0–0.7]	23 (4.8)	25 (2.9)
Village					
Nyamisati	213 (24.9)	0 (0.0) [0.0–1.8]	0 (0.0) [0.0–1.8]	7 (4.1)	5 (2.3)
Nyambili	196 (23.0)	6 (3.1) [1.4–6.5]	1 (0.5) [0.1–2.8]	5 (5.0)	10 (5.1)
Nyanjati	189 (22.1)	0 (0.0) [0.0–2.0]	0 (0.0) [0.0–2.0]	3 (3.6)	7 (4.1)
Bungu	172 (20.1)	3 (1.7) [0.6–5.0]	0 (0.0) [0.0–2.2]	4 (5.1)	3 (1.7)
Mchukwi	84 (9.8)	0 (0.0) [0.0–4.4]	0 (0.0) [0.0–4.4]	4 (8.3)	0 (0.0)
Total	854	9 (1.1) [0.6–2.0]	1 (0.1) [0.0–0.7]	23 (4.8)	25 (2.9)

^a^Denominator included only males

### LF transmission indices

A total of 3334 mosquitoes were caught using CDC light traps of which 69 (2.1%) were identified as species of the *Anopheles gambiae* complex, 1054 (31.6%) were *Culex quinquefasciatus*, and 2211 (66.3 %) were non-filarial vector species. In the laboratory, filarial mosquito vectors were dissected for parity, and 558 parous vectors were examined under microscopy for infection with *W. bancrofti*. None of the mosquitoes examined for infection was found to carry *W. bancrofti* larvae of any stage (Table 3).

**Table 3:** Filarial mosquito vectors caught and analyzed for infection and or/infectivity with *W. bancrofti* using microscopy and qPCR

Collection method	Vector collected	Parous vectors	Method of analysis	No. analyzed	No. infected (%)	No. infective (%)
Light trap	1123^a^	558^b^	Microscopy	558	0 (0)	0 (0)
Gravid trap	5460	–	qPCR	1092^c^	5 (0.1)^d^	0 (0)

^a^Filarial vector composition: *Anopheles gambiae* complex (*n* = 69) and *Culex quinquefasciatus* (*n* = 1054)

^b^Parous vectors: *An. gambiae* complex (*n* = 45) and *Cx. quinquefasciatus* (*n* = 513)

^c^Pools of 5 *Cx. quinquefasciatus* each

^d^Infection rate (Pool Screen V2.0.2; Likelihood ratio method; 95% CI: 0.03–0.22%)

A total of 5460 gravid *Cx. quinquefasciatus* were collected outdoors using CDC gravid traps and dissected to separate head, thorax and abdomen. An aliquot of DNA from the head, thorax and abdomen of the 5460 *Cx. quinquefasciatus* was tested in pools of five mosquitoes (a total of 1092 pools) with qPCR. Of 1092 pools of *Cx. quinquefasciatus* tested, five pools were positive for *W. bancrofti* DNA. Further analysis by pool screen (v2.0.2 software) gave an estimate that 0.1% of the tested mosquitoes were infected (Table 3). Further analysis of an aliquot of DNA from head segments of the infected pools revealed the absence of infective mosquitoes (i.e. mosquitoes harboring third-stage larvae of *W. bancrofti*).

### MDA compliance concerning LF infection status

Of the 854 individuals interviewed, 82.4% reported having participated in at least one previous MDA round, while 70.6% participated in an MDA that was conducted in September 2014 (about seven months before the present study). Individuals who did not participate in any of the previous rounds of MDA were significantly more infected (CFA) compared with those with a recent history of participating in MDA (*χ*^2^ = 8.723, *df* = 1, *P* = 0.003). The proportion of males with hydrocele was significantly higher in individuals who did not swallow drugs in any MDA round while the prevalence of elephantiasis was not significantly different between these two groups of participants (Table 4).

**Table 4:** Demographic characteristics of the study population and their reported drug uptake in relation to their LF infection status

Characteristic	No. (%) of total	No. (%) with CFA	*χ*^2^-value (*P*-value)	No (%)^a^ with hydrocele	*χ*^2^-value (*P*-value)	No (%) with elephantiasis	*χ*^2^-value (*P*-value)
Gender (*n* = 854)							
Males	481 (56.3)	8 (1.6)	3.921 (0.048)	23 (4.8)	–	18 (3.7)	2.573 (0.109)
Females	373 (43.7)	1 (0.3)				7 ( 1.9)	
Age groups in years (*n* = 854)							
≤ 29	450 (52.7)	4 (0.9)	0.248 (0.618)	5 (2.0)	8.723 (0.003)	13 (2.9)	0.005 (0.944)
≥ 30	404 (47.3)	5 (1.2)		18 (7.8)		12 (3.0)	
Reported drug uptake in previous MDAs (*n* = 854)							
Yes	704 (82.4)	4 (0.6)	8.723 (0.003)	9 (2.4)	22.743 (0.001)	19 (2.7)	0.737 (0.391)
No	150 (17.6)	5 (3.3)		14 (13.7)		6 (4.0)	
Reported drug uptake in 2014 MDA (*n* = 854)							
Yes	603 (70.6)	6 (1.0)	0.068 (0.794)	8 (2.5)	10.243 (0.001)	16 (2.7)	0.542 (0.462)
No	251 (29.4)	3 (1.2)		15 (9.1)		9 (3.6)	

^a^Denominator included only males: for age group ≤ 29 and ≥ 30 there were 249 and 232 males; drug uptake in any previous MDA, 379 males swallowed the drugs while 102 did not swallow the drugs; drug uptake in 2014 MDA, 316 males swallowed the drugs while 165 did not

## Discussion

LF elimination activities based on annual MDA with a combination of ivermectin and albendazole have been ongoing in Tanzania for more than a decade. Thus, regular monitoring is essential to evaluate the progress and make informed decisions on when it is safe to stop MDA intervention. Studies on the impact of MDA on LF transmission and infection in areas implementing diethylcarbamazine (DEC) and albendazole regimes such as American Samoa, India, Egypt and Papua New Guinea, have documented good progress towards elimination [23–25]. In sub-Saharan Africa, many LF-endemic countries are co-endemic with onchocerciasis and thus ivermectin is used to avoid the potential DEC-induced side effects on onchocerciasis patients. Using a combination of ivermectin and albendazole MDA strategy, some West African countries are showing good progress toward elimination of LF [26, 27], and Togo has been declared to have achieved LF elimination target [28, 29]. In northeastern Tanzania, the impact of MDA with this drug combination has shown a remarkable decline in LF infection and transmission [30]. The documented declining trend in LF transmission suggests the need to conduct a WHO-approved transmission assessment survey (TAS) to determine whether MDA can be stopped in endemic areas showing good progress.

The findings of this study have shown that after 12 rounds of ivermectin and albendazole MDA in the Rufiji District there has been a progressive decline in LF prevalence and transmission since the start of MDA intervention in 2002. Before the MDA intervention, the prevalence of CFA, MF, hydrocele and elephantiasis was 49%, 18%, 12% and 4%, respectively (MoH 2000, unpublished). In this study, no children tested positive for CFA in 2015 surveys (after 12 rounds of MDA). This shows a significant decline in CFA rates compared to our previous findings in the same study area in 2012 (after 9 MDA rounds), which showed CFA rate of 14.3% among pupils screened [31]. This decline in CFA suggests a substantial reduction in the acquisition of new infection in children from 2012 to 2014. The prevalence of MF and CFA recorded in the current study (0.1% and 1.1%, respectively) were below the WHO-recommended elimination thresholds of 1% (for MF) and 2% (for CFA) [3]. Our findings on the decline of LF infection and transmission due to MDA intervention corroborate those reported by Simonsen et al. [30] in northeastern Tanzania.

Detection of *W. bancrofti* infection in mosquito vectors is an essential aspect of monitoring LF transmission as it provides real-time information on the presence of local transmission [14]. CDC light and gravid traps have been considered efficient tools for the collection of filarial vectors in areas where *Anopheles* and *Cx. quinquefasciatus* are the primary vectors [32]. Important filarial mosquito vectors found elsewhere in Tanzania, such as the species of the *An. gambiae* complex, *An. funestus* group and *Cx. quinquefasciatus* have also been reported in Rufiji District [33]*.* In this study, none of the dissected filarial vectors examined for infection (*Cx. quinquefasciatus* and *An. gambiae* complex) were found to carry *W. bancrofti* larvae of any stage. Using qPCR with high throughput and precision, the probability of finding an infected mosquito in any of the analysed pools of gravid *Cx. quinquefasciatus* was estimated at 0.1%. Importantly in the transmission, none of the mosquitoes tested with qPCR was found to carry an infective stage of *W. bancrofti*. The fact that we did not identify any LF-infective vector by either qPCR or dissection suggests that local transmission is extremely low if occurring at all in the study areas.

The present study showed that the prevalence of hydrocele and elephantiasis was lower than that reported by the MOH before MDA (MoHSW 2000, unpublished), but the survey methods differed and a statistical comparison of prevalence in the two studies could not be made. In contrast, the proportion of individuals with elephantiasis in the present study was similar to that reported by Gasarasi et al. [34] who conducted a study in the same area in 2000. Other studies conducted elsewhere have indicated a moderate to no reduction in the prevalence of lymphoedema after several rounds of MDA [35, 36]. Therefore, the observed low rate of hydrocele in this study might be explained by the ongoing hydrocelectomy intervention in the district.

Treatment coverage and compliance are crucial factors to observe when assessing the impact of MDA on LF transmission. For MDA strategy to be effective, sustained high treatment coverage is critical to reach the elimination target within a reasonable time-frame [37]. Moreover, empirical evidence suggested that endemic areas with high baseline infection levels will require higher treatment coverage and a more sustained MDA intervention [38]. However, it has been documented that attaining optimal drug uptake during MDA is a challenge in LF control programmes in most of the endemic areas [39–41]. In this study, the surveyed drug uptake was 70.6 and 82.4% for the last MDA round (September 2014, seven months before the present study) and participation in any previous MDA rounds, respectively. The surveyed MDA coverage in our study was at the recommended range of 60–70% drug uptake rates required for interruption of transmission [37]. Although drug uptake was found to be significantly lower in respondents with hydrocele, previous studies have reported a low prevalence of microfilaraemia in individuals with hydrocele and elephantiasis [42, 43].

Our study has recorded a progressive and substantial decline in LF prevalence in the study areas when compared to the baseline values before MDA and also to a more recent survey conducted in the same villages in 2012 [31]. In addition to the impact of MDA, the universal distribution of long-lasting insecticide-treated nets which was implemented in 2012 could contribute to the drastic decline in LF disease prevalence. Insecticide-treated nets distributed for malaria control have been shown to reduce LF prevalence in some settings [44–47].

Despite a substantial decline in LF in this study areas, a recommendation to stop MDA could not be made because the current study methods did not follow WHO-approved TAS. The design of the present study was adopted as a result of limited funding and time to implement an elaborated WHO-approved TAS protocol. Moreover, the direct statistical comparison between the baseline study in 2000 and the present study could not be made due to the unavailability of a detailed methodology for the baseline study. With these limitations, it is advisable that a formal TAS is conducted before MDA can be stopped the area.

## Conclusions

Analysis of indices of LF infection in the human population and filarial mosquito vectors demonstrated a substantial decline in the prevalence of LF in the study areas when compared with baseline values before the start of elimination activities based on MDA. We recommend a formal TAS to be conducted in the study areas to make an informed decision on whether MDA can be stopped in Rufiji District.

## Abbreviations

LF: Lymphatic filariasis
MDA: Mass drug administration
CFA: Circulating filarial antigens
ICT: Immunochromatographic test cards
qPCR: Quantitative real time polymerase chain reaction
TAS: Transmission assessment survey
WHO: World health organization
CDC: Centers for Disease Control and prevention
DNA: Deoxyribonucleic acid
GPELF: Global Programme to Eliminate Lymphatic Filariasis
NLFEP: National Lymphatic Filariasis Elimination Programme
